# Neural Network Clustering and Swarm Intelligence-Based Routing Protocol for Wireless Sensor Networks: A Machine Learning Perspective

**DOI:** 10.1155/2023/4758852

**Published:** 2023-07-26

**Authors:** Awatef Salem Balobaid, Saahira Banu Ahamed, Shermin Shamsudheen, S. Balamurugan

**Affiliations:** ^1^Department of Computer Science, College of Computer Science & Information Technology, Jazan University, Jazan 45142, Saudi Arabia; ^2^Bule Hora University, Ministry of Education, Oromia, Ethiopia

## Abstract

With no requirement for an established network infrastructure, wireless sensor networks (WSNs) are well suited for applications that call for quick network deployment. Military training and emergency rescue operations are two prominent uses of WSNs. The individual network nodes must carry out routing and intrusion detection because there is no predetermined routing or intrusion detection in a wireless network. WSNs can only manage a certain volume of data, and doing so requires a significant amount of energy to process, transmit, and receive. Since sensors have a modest energy source and a constrained bandwidth, they cannot transmit all of their data to a base station for processing and analysis. Therefore, machine learning (ML) techniques are needed for WSNs to facilitate data transmission. Other current solutions have drawbacks as well, such as being less reliable, more susceptible to environmental changes, converging more slowly, and having shorter network lifetimes. This study addressed problems with wireless sensor networks and devised an efficient clustering and routing algorithm based on machine learning. Results from simulations demonstrate that the proposed system beats previous state-of-the-art models on a variety of metrics, including accuracy, specificity, and sensitivity (0.93, 0.93, and 0.92 respectively).

## 1. Introduction

In certain real-time applications, wireless sensor networks (WSNs) [1] offer advantages, such as size, cost-effectiveness, and easy deployment that make them promising technologies [2]. Sensor nodes are employed in large numbers in WSN applications to monitor a field and gather data that are then sent to the base station for processing [3, 4]. As a result, controlling such a vast number of nodes necessitates the development of a scalable and efficient algorithm. WSNs can also change dynamically due to external factors or according to the design plans of the system developers. As a consequence, routing methods, localization, latency, cross-layer design [5], coverage, quality of service, fault detection, link quality, and other aspects of the system may be affected [6]. Depending on the dynamic network, it may be necessary to depreciate nonessential configurations. However, traditional WSN systems are explicitly coded, and as a result, they cannot be correctly implemented in a dynamic environment. In machine learning (ML), improvements or learning occur without explicit programming based on knowledge or studies [7, 8].

**Figure 1:**
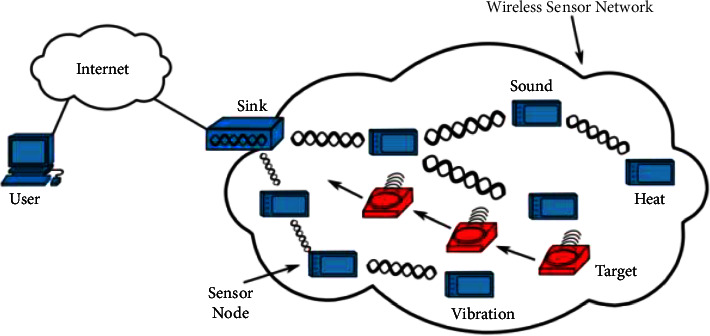
Wireless sensor network structure [9].

By using machine learning, machines became more reliable, more accurate, and less expensive. Models are created using machine learning, allowing for more complex data to be analyzed automatically, swiftly, and precisely. ML [9–12] is well known for its ability to provide generic resolutions using an approach that learns to improve its efficiency over time. As a result, supervised learning, semisupervised learning, unsupervised learning, and reinforcement learning are all part of machine learning. WSN difficulties have been solved using recent improvements in machine learning [1]. Because of their transdisciplinary nature, WSNs are important for engineering, medicine, and computers (Figure 1). They assist in gaining access to and obtaining useful information from the vast quantities of data produced by sensors without requiring human intervention or reprogramming. WSNs' performance improves via machine learning, which eliminates the necessity for human interference or reconfiguration. They use cloud computing and big data processing in addition to the Internet of Things (IoT) and machine-to-machine integration [1].

### 1.1. Challenges in WSNs and Machine Learning Techniques

Normally, sensor nodes are positioned in dangerous regions where the network is left to operate autonomously without human intervention. When constructing WSNs, researchers must take into consideration factors such as battery power limitations, memory constraints, connection failure, dynamic topology changes, and decentralized control. In this section, we look at how machine-learning techniques may be used to address various problems in wireless sensor networks. Three categories are used to group WSN challenges. Focus should be placed on three primary areas: functioning, future challenges, and security and other issues [13]. Below, each of these problems is discussed.

#### 1.1.1. Functional Challenges

Additionally, various machine learning algorithms address clustering and data aggregation, energy harvesting, MAC management, location, event detection and query processing, mobile sink, object tracking, traffic regulation, coverage, and network issues, while traditional routing systems handle routing issues.

#### 1.1.2. Aggregation and Clustering of Data

The direct delivery of sensed data to the sink node is a need in WSNs in large-scale networks. The use of clustering can allow for the direct transmission of data to the sink node, resulting in considerable energy savings. Additionally, selecting efficient cluster heads can contribute to the use of low-energy clustering. Cluster nodes in a cluster head (CH) gather data from each other.

#### 1.1.3. Query Processing and Event Detection

It is a common requirement in many WSN applications that the user detects activity around moving objects and relays this back to the user. In addition, these activities usually take place in different places and last an indeterminate period. WSNs may be observed in three ways: event-driven, continuous-monitoring, and query monitoring. With limited resource capability, machine learning may assist in delivering effective query processing and solutions, thus identifying events, and analyzing event authenticity. It is still necessary to create improved event detection and query processing algorithms utilizing various machine learning approaches. Although the academic community has taken an active interest in these areas [14], there is still a gap.

#### 1.1.4. Energy Harvesting

Using solar energy to power sensor nodes in an open environment has emerged as a viable option for providing the nodes with enough power in a long-term manner. It is the process of converting energy from the environment into electrical energy. Several obstacles may arise in the effective integration of energy harvesting technologies into WSNs [15–20].

#### 1.1.5. Object Tracking and Localization

Localization is a procedure for detecting the global position of a sensor node utilizing GPS technology, which is prohibitively expensive for large-scale networks. Most WSN applications need to locate their sensor nodes geographically, which becomes an issue.

#### 1.1.6. Mobile Sink

When sensors collect data through a WSN, the data are transmitted to sink nodes in an isolated or distributed manner. In the vicinity of the sink nodes, the nodes become bottlenecks, resulting in hotspots. Thus, mobile sinks have emerged as a concept in WSN research. The information is collected by the mobile sink as it moves from one sensor node to the next. Mobile sinks gather information from rendezvous points rather than visiting each sensor node. Since it is difficult to visit every sensor node, scheduling the mobile sink is a research focus. The information is sent to RPs via other sensor nodes. To eliminate delays, numerous mobile sinks are sometimes employed, although this is not cost-effective. Using a mobile sink, ML can aid in discovering optimum RPs and avoiding delays.

#### 1.1.7. Congestion Control

In WSNs, congestion can occur when data quantities exceed the available bandwidth. This happens when the data link transmits more data than the channel can handle. As congestion is usually found at the connections or nodes level, it affects latencies, packet losses, and quality of service, as well as total energy consumption. ML algorithms can accurately predict traffic, remove end-to-end delays, and dynamically modify transmission ranges based on packet arrival rates at nodes and bit error transmission rates at links. Lower packet error rates and collisions between nodes can cause congestion at the node and link levels.

#### 1.1.8. Coverage and Connectivity

In WSNs, coverage and connection are key issues. WSNs are often deployed in a deterministic or random manner in a given area. It is connected with a BS station or relay node if it can communicate directly with the BS station or relay node. A sensor node's coverage is the extent to which it is able to monitor a particular area. The nodes will have a gap if there are not enough sensors in the region. As connectivity problems arise, ML can be used to determine how many sensors are needed to cover the target region and adjust the route dynamically as necessary.

Since the nodes of wireless sensor networks (WSNs) solely run on battery power, energy saving is one of the main issues with these networks. A network of sensor nodes serves as the foundation of a WSN design. The development of effective and reliable communication protocols is essential for overcoming WSN challenges and maintaining the network for a long time. Traditional technologies can solve a lot of WSN problems, but they might not be able to produce a good enough mathematical model to anticipate network behavior. In order to characterize system behavior for challenging tasks like routing, data fusion, localization, and object tracking, low-complexity mathematical models are used. With the help of machine learning techniques, this study aims to create a cluster and routing protocol for WSNs that takes into account concerns such as battery life, adaptability to environmental changes, reliability, convergence speed, and network lifetime. The amount of energy required for data gathering and distribution is drastically reduced by using hierarchical routing protocols based on swarm intelligence in conjunction with neural network-based clustering techniques. Using a variety of important criteria, this technique is evaluated against several clustering and routing strategies.

### 1.2. Key Highlights

The goals of this study, which focuses on improving WSN routing, are as follows:

- Create a clustering and routing mechanism for WSNs based on ML
- Create a reliable and effective model to address WSN challenges and sustain the network for a long time
- Neural network-based and swarm intelligence-based techniques are utilized, respectively, for clustering and routing
- According to the experimental analysis, the suggested model performs better and uses less energy in WSNs

Organization of the paper: We provided an overview of a WSN and its challenges in Section 1. The rest of the paper is as follows: Section 2 presents a literature review, Section 3 describes the overall methodology, Section 4 depicts the performance analysis, and finally, the paper is concluded in Section 5.

## 2. Related Works

As many researchers attempt to investigate the field of wireless networks and their integration with machine learning for future purposes, some research is organized into categories, such as classical-based machine learning, optimization-based machine learning, and neural network-based machine learning.

### 2.1. Classical-Based Techniques

To calculate the number of clusters, traditional approaches employ a statistical technique. To replace a CH with traditional methods, a threshold is built into a method that assesses the number of nodes with remaining energy or their energy consumption, which causes the network to recluster [16]. In cluster-based routing protocols, traditional techniques focus on how to pick cluster heads. Pal Singh and Chander Sharma [17] proposed a method for improving the routing protocol. The Voronoi diagram is then generated with the cluster head as the center based on the predictions made in accordance with the estimated total energy usage. Cluster heads are then calculated every round according to the predicted total energy usage. This reduces the energy consumed for intracluster communication, as the nodes in the Voronoi diagram form a cluster. The multihop routing protocol uses an ant colony algorithm to optimize the node-to-node routing by having a cluster head located near the BS that accepts and routes data from the cluster's nodes. MATLAB simulations indicate that the protocol is superior to LEACH in extending the lifetime of WSNs, as well as in improving energy efficiency per node each round. As Ahmad et al. (2015) [18] discovered, by considering the intracluster distance, intercluster communication, and the farthest cluster head (CH) for a cluster rather than the closest cluster head (CH) for the cluster, the lifetime of a network can be extended.

### 2.2. Optimization-Based Techniques

Shokouhifar and Jalali [19] provided a method for determining the path from each cluster's CH to BS. Once the firefly is switched on, it provides an alternate route from the CH to the BS. Each firefly is the same size as the number of CHs with an additional spot for the BS. Each point in the CH's routing data to the BS represents its next hop. Every time the lower-brightness firefly approaches the higher-brightness firefly, each firefly's position is updated. The procedure is repeated until the conditions are met. In the fitness function, the energy for the next residual hop, the degree of a node, the distance between the CH and the next hop, and the number of CH members making up the next hop are considered.

Rao [20] proposed a particle swarm-based optimization clustering approach with a mobile sink for wireless sensor networks. This algorithm employs the virtual clustering strategy throughout the routing step, which utilizes the particle swarm optimization algorithm. Residual energy and node location are the most important factors to consider when selecting a cluster head. The control approach is used by the mobile sink to collect data from the cluster head. Comprehensive simulated results show that our proposed routing technique consumes less energy, has a longer network lifetime, and has a lower transmission delay than several existing commonly used routing methods.

Radhika and Sivakumar [21] suggested an enhanced artificial bee colony optimization-based clustering technique (IABCOCT) that ensures optimal clustering and CH selection by combining the advantages of the grenade explosion and the Cauchy operator. This provides a lot of investigation and enhancement to the level of analysis and exploration in the observer bee and scout bee phases, which helps determine the optimal cluster heads. Using this clustering process improves the cluster head selection accuracy while also lowering the node energy consumption.

### 2.3. Neural Network-Based Techniques

Using SVM as the goal, Anand et al. [22] designed a decision function based on statistical learning theory. This decision function has a simple implementation at the cluster nodes to detect abnormal sensors since it allows for low-resolution detection. Compared to the newest fault detection algorithm for WSNs, SVM is more effective at detecting faults in WSNs through an experimental investigation.

The synthetic minority oversampling technique (SMOTE) suggested by Nayak et al. [23] is used to balance the dataset before training the intrusion detection classification through the random forest algorithm. Random forest accounts for 92.39% of the accuracy in a benchmark intrusion dataset, which is higher than that of other similar algorithms. Additionally, random forest paired with the SMOTE has grown the accuracy to 92.57% after oversampling the minority samples.

Using multilayer perceptron neural networks (MLPNN) and radial basis function neural networks (RBFNN) to construct an analysis framework for localization in WSNs, Raj [24] compared and analyzed them. As a result, the received signal strength indicator (RSSI) from three anchor nodes with fixed placements was used to determine whether the static sensors were within the 100 100 m^2^ grid. Simulation results suggested that the MLPNN performs better than the RBFNN.

## 3. Clustering and Routing Protocol Using ML

### 3.1. Data Transmission Model

**Figure 2:**
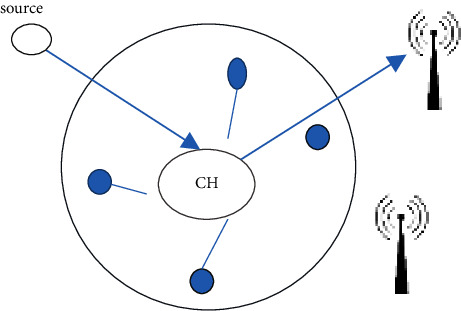
Transmission of data.

At first, sensor nodes are scattered randomly across a sensor field and powered by full-capacity batteries. The network is made up of sensor nodes with limited power. The data gathered by each sensor are typically linked with the data of surrounding sensors, resulting in the delivery of data relating to each sensor to the BS for evaluation or consideration for making choices. We consider periodic sensing with the same time interval for all sensors. We employ a fixed clustering mechanism that leads to cluster selection as a result of a novel clustering approach [25]. Within each cluster, a node is regularly chosen to serve as a communication hub (CH), enabling communication to and from the cluster (Figure 2).

### 3.2. Model for Attaining Energy

A radio transceiver's energy consumption is estimated by using the following energy model [26]. Basically, a transmitting node sends each packet to one or more receivers within its vicinity and then calculates how much of that energy is consumed by each node.

(1) $$\begin{array}{c} C=C_{t}+{nC}_{r}+\left(N-n\right)C^{h}r\text{.} \end{array}$$

Because *C*_*t*_ and *C*_*r*_ are dependent on the number of neighbors who must receive the data, *n* is the number of neighbors who must be included in the transmission, and *C*_*t*_ and *C*_*r*_ are the energy required to transmit and receive the packet, respectively. According to the paradigm provided in [27], *C*_*t*_ or *C*_*r*_ denotes the amount of energy necessary for only decoding the packet header for a distance *d* and a *k*-byte message.

(2) $$\begin{array}{c} C_{t}\left(d,k\right)=\left(C_{elect}+C_{amp}*d_{\rho }\right)8k\text{,} \end{array}$$

(3) $$\begin{array}{c} C_{r}\left(k\right)=C_{elect}*8k\text{.} \end{array}$$

### 3.3. Clustering Using NN

We approach the energy-aware routing problem via an LP formulation. Its goal is to identify the node with the greatest amount of remaining energy and include it in the best path while minimizing network costs. Assume that the CH nodes range from 1 to *n*, where *n* is the number of CH sensor nodes, and that the base station node is 0.

(4) $$\begin{array}{c} {Minimize\sum }_{1\leq i\leq n}R_{-}C\text{,} \end{array}$$

constrained by the following

(5) $$\begin{array}{c} \underset{1\leq j\leq n}{\sum }D_{ij}-\underset{1\leq j\leq n}{\sum }D_{ji}=b_{i}\cdots \cdots \\ D_{ij}\geq 0,1\leq j\leq n\\ E\leq P_{maximum}\cdots \cdots \cdots \cdots \cdots P_{i}=\left[x_{t,},x_{l,2},x_{i,3}\cdots ,x_{i,D}\right]\text{.} \end{array}$$

The first and second constraint sets the maximum quantity of data that can be shared among two nodes, *S*_*i*_ and *S*_*j*_, while the third ensures that each node in the network has a minimal lifespan and controls the maximum power usage [14, 28–30].

#### 3.3.1. Setup Phase

Here, an energy-conscious path is built using a swarm intelligence algorithm based on cluster selection and cluster creation.

#### 3.3.2. Cluster Head Selection

To prevent the network from being overloaded with energy consumption throughout the network lifecycle, several clustering algorithms provide evenly dispersed clusters with constant average cluster sizes. However, we present a cutting-edge neural network-based coverage-aware clustering method [31]. Equation (3) allows for the selection of cluster head nodes. Cluster head nodes are congested in the network's more populated areas, but not in its sparsely populated ones. Given that the proposals include three layers—the input layer, the competition layer, and the output layer—a neural network with three layers attempts to further decrease the lifetime of expensive sensors in sparsely covered areas.

**Figure 3:**
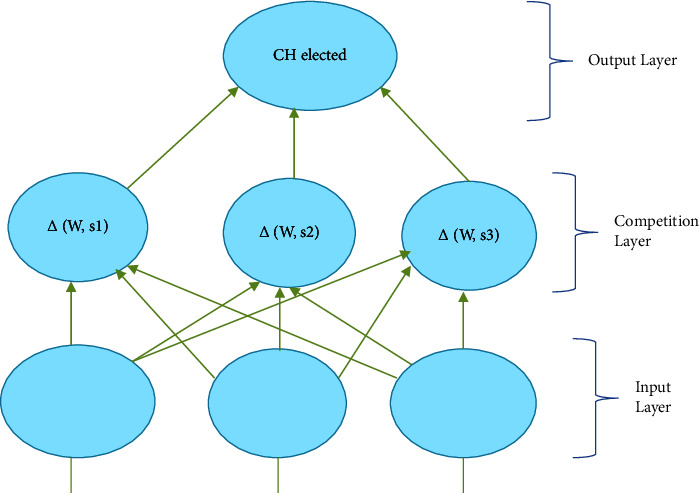
Cluster head formation using NN.

They have a number of benefits, including flexibility, simplicity, parallelization, speed, and adaptability. They can learn and have been applied to a range of problems. The competitive learning theory is applied to a two-layer feedforward neural network in Figure 3. The sensor nodes that compete for CH provide input patterns to the input nodes. Each output node is coupled with a cluster and has a weight *W*_*j*_, *j* = 1, 2,…, *m*, where *m* is the number of clusters. It is suggested that the CH selection technique involves adaptive learning in each neuron in the competitive layer because only the neuron with the lowest *E*_*i*_^*D*^ value succeeds in getting activated or firing. Learning is determined by the learning pace, which directly affects convergence. If it is set to 0, there is no learning. The prototype vector is given to the input if it is set to one. Between the preceding vector value and the remaining input pattern selections, a new vector position is created. In general, the learning rate might be constant or fluctuate (Algorithm 1).

### 3.4. Routing Using Swarm Intelligence

It may be used to represent the *n*th particle (*P*_*i*_) in the *n*-dimensional population as a function of a bird's or fish's behavior in a group. Particles adjust their location in response to a group's position and velocity. In the case of an *n* D-dimensional population, the *i*th particle (*P*_*i*_) is represented as follows:

(6) $$\begin{array}{c} P_{i}=\left[x_{L,1},x_{i,2},x_{t,3}\cdots ,x_{i,D}\right]\text{.} \end{array}$$

A fitness function is applied to every object's location to evaluate the accuracy of the response it supplies in the present iteration. Tracking the particle's personally best position (*P*best) and the particle's overall best position (*W*best) can provide the particle with its globally best position (*G*best). *V*_id_ and *X*_id_ can be modified, and the velocity and location of each particle are as follows:

(7) $$\begin{array}{c} V_{new,i}=w*V_{i}+c_{1}*r_{1}*\left(Xpbest_{i}-X_{i}\right)+c_{2}*r_{2}*\left(X_{gbest}-X_{i}\right)\text{.} \end{array}$$

The weight of inertia can be determined using the following formula, where inertia weight is *w*, *c*_1_ and *c*_2_ are accelerating coefficients, and *r*_1_ and *r*_2_ are arbitrary variables in the [0, 1] range.

(8) $$\begin{array}{c} w=w_{initial}-\frac{\mathrm{Max}.Iteration-Current\;Iteration}{Total\;Number\;of\;Iterations}X_{new,i}=X_{old,i}+V_{new}\text{.} \end{array}$$

The ideal number of CHs is generated using a suggested PSO method. The suggested PSO algorithm's problem is a time-varying equation with a new value for each iteration. Furthermore, if a sensor node is within its communication range, it can be used as a cluster head. The essential knowledge paradigm is similar to traditional algorithms, such as LEACH. Every new cycle begins with information sent from each node to the cluster head. As soon as the information is collected by the cluster head, repeated data are deleted, and the actual data are sent to the subsequent hop, which is generally the base station or another cluster head. (Algorithm 2)

## 4. Results

The proposed model (NNC-PSOR) is compared to state-of-the-art models, such as WCDT, FCM, LEACH, PEACH, ANFIS, LEACH-GA, and FBR on key parameters such as network lifetime, clustering speed, accuracy, quality, sensitivity, randomness in results, performance, energy management, and security.  Table 1 shows the simulation network lifetime based on the number of iterations (6000) based on different models. We employ a 400 400 pixel wide, 50 m transmission range, stationary wireless sensor network. A message should be 48 bytes long, including the packet header of 12 bytes. The system is constructed using the MATLAB program on a computer with a core 2duo CPU running at 1.6 GHz.

**Figure 4:**
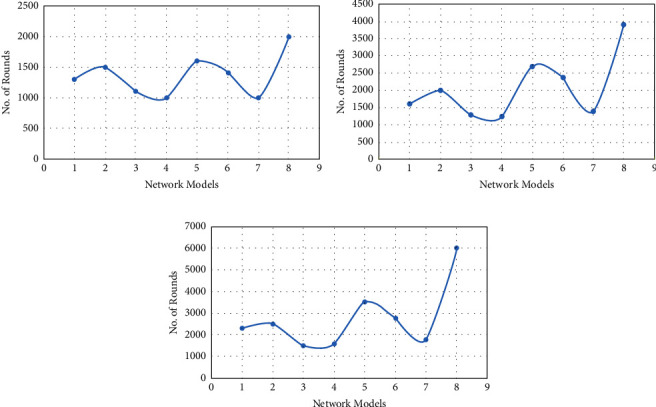
Network models vs. no. of rounds over (a) first-node dies, (b) half-node dies, and (c) last-node dies.

Figure 4(a) shows a graphical depiction of numerous state-of-the-art models across a number of iterated rounds, including WCDT (1), FCM (2), LEACH (3), PEACH (4), ANFIS (5), LEACH-GA (6), FBR (7), and NNC-PSOR (8). On each parameter, our proposed strategy (NNC-PSOR) outperforms LEACH, PEACH, and other existing approaches by more than 90%.

**Figure 5:**
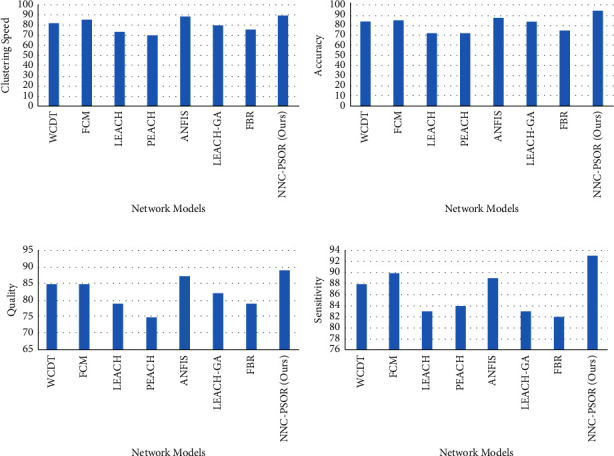
(a) Network models vs clustering speed, (b) network models vs accuracy, (c) network models vs quality, and (d) network models vs sensitivity.

**Figure 6:**
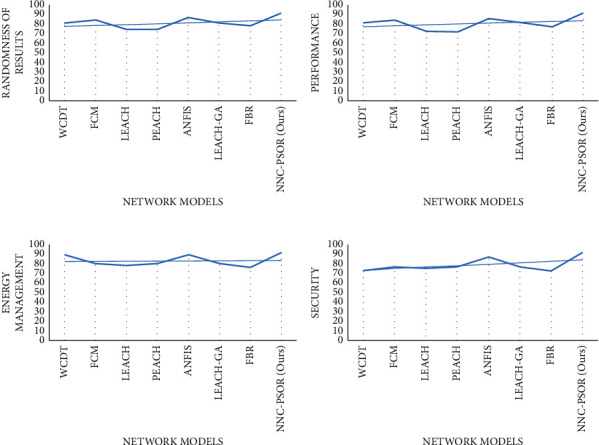
(a) Network models vs randomness of results, (b) network models vs performance, (c) network models vs energy management, and (d) network models vs security.

Figure 5 shows a graphical representation of model attributes, such as clustering speed, accuracy, quality, and sensitivity. The graphical depiction of several models with parameters such as unpredictable results, performance, energy management, and security is shown in Figures 6(a)–6(d).

## 5. Discussion

Based on simulations of all models for 6000 rounds, FBR and PEACH each die at 1000, while the proposed NNC-PSOR and ANFIS die at 1600 and 2000.  Figure 4(b) displays a graphical depiction of numerous state-of-the-art models across a number of iterated rounds, including WCDT (1), FCM (2), LEACH (3), PEACH (4), ANFIS (5), LEACH-GA (6), FBR (7), and NNC-PSOR (8). When all models are run for 6000 rounds, half of the nodes die at 1250 and 1300 for LEACH and PEACH, respectively, compared to 2700 and 3900 for ANFIS and NNC-PSOR (Ours).  Figure 4(c) shows a graphical depiction of numerous state-of-the-art models across a number of iterated rounds, including WCDT (1), FCM (2), LEACH (3), PEACH (4), ANFIS (5), LEACH-GA (6), FBR (7), and NNC-PSOR (8). When all models are run for 6000 cycles, the first node for LEACH and PEACH dies at 1500 and 1590, respectively; comparatively, models such as ANFIS and NNC-PSOR die at 3500 and 6000.

## 6. Conclusion

This paper demonstrates the usefulness of clustering and routing using machine learning in this study, resulting in a considerable increase in the network lifetime and performance of WSNs. When analyzing a network via simulation compared to existing models, the integration of neural networks and particle swarm optimization allows for much longer network life. As technologies are being revolutionized in an advanced way, the integration of several models instead of going in one direction can increase performance. However, machine learning algorithms cannot automatically make reliable predictions, as they must learn from historical data. Other researchers are encouraged to expand on this work to develop even more effective clustering and routing methods for WSNs. Increasing the size of the information also increases the amount of energy used to process the data, which then affects the performance. With the simulation results, swarm intelligence outperforms standard techniques under a variety of network conditions, providing a high degree of flexibility and efficiency. In order to further examine the performance, real-time data are required, which is one of the restrictions. The application of this model with current and real-time data will be required in the future to further boost efficiency in order to get beyond these restrictions.

## Figures and Tables

**Algorithm 1:**
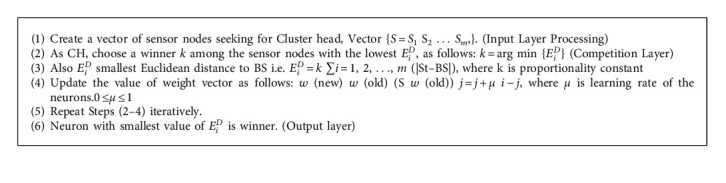
Algorithm for CH selection using NN.

**Algorithm 2:**
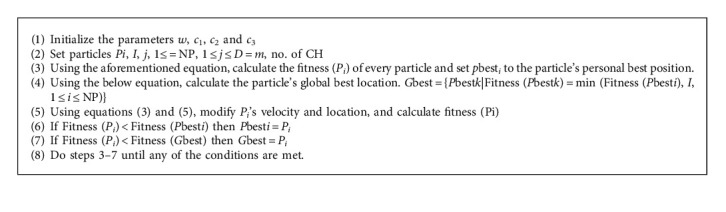
Algorithm for routing using PSO.

**Table 1:** Simulation results of the network lifetime.

Network models	First-node die	Half-node die	Last-node die
WCDT	1300	1600	2300
FCM	1500	2000	2500
PEACH	1000	1250	1590
LEACH	1100	1300	1500
ANFIS	1600	2700	3500
LEACH-GA	1400	2380	2750
FBR	1000	1390	1780
NNC-PSOR	2000	3900	6000

## Data Availability

The datasets used and/or analyzed during the current study are available from the corresponding author on reasonable request.
